# Supplementary figures and images for: Gene Regulation during Carapacial Ridge Development of Mauremys reevesii: The Development of Carapacial Ridge, Ribs and Scutes
Source: Genes (Basel). 2022 Sep 19;13(9):1676. doi: 10.3390/genes13091676 (PMC9498798; doi:10.3390/genes13091676)

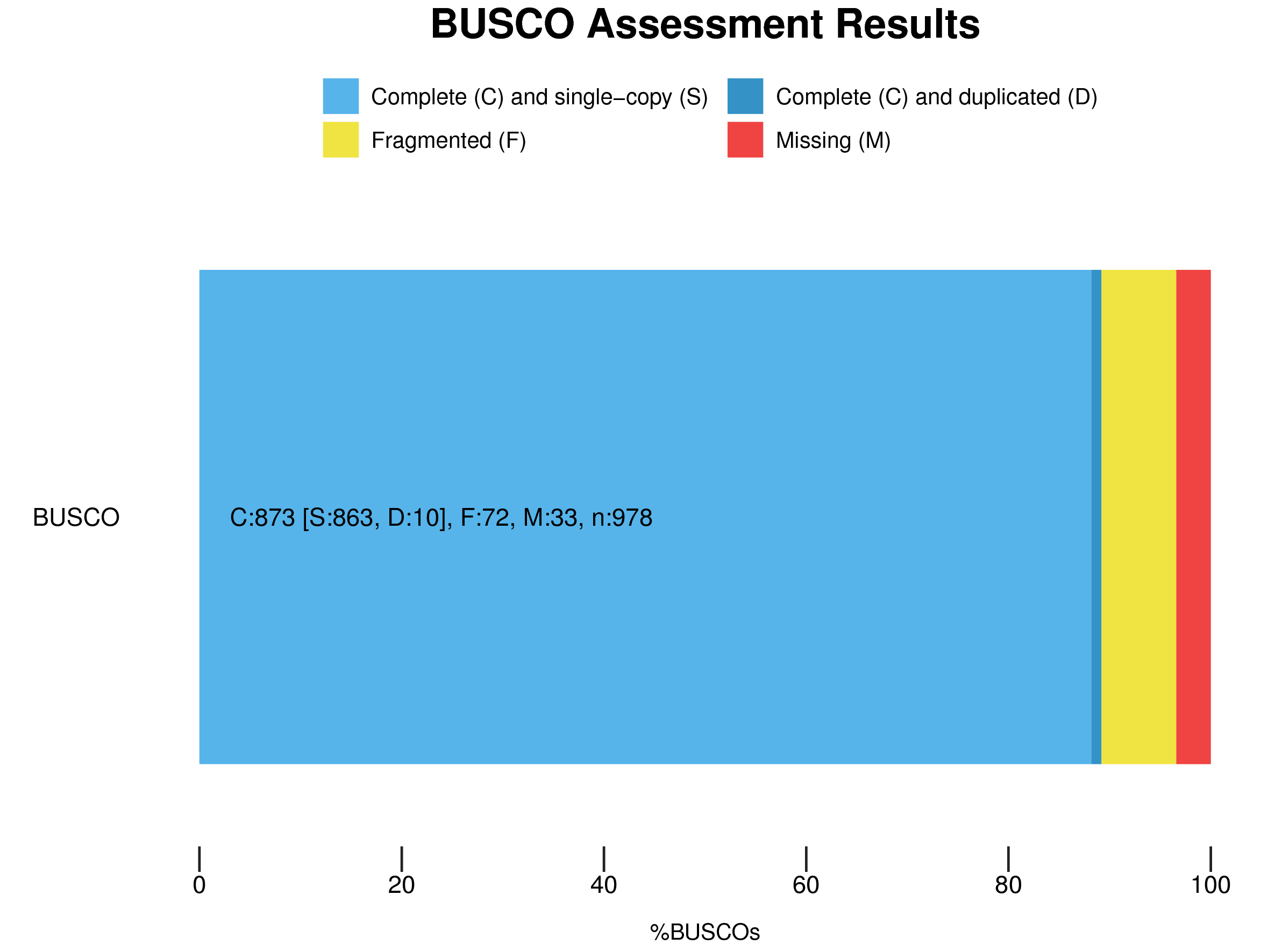

Supplement: Supplementary file 1 [file genes-13-01676-s001.zip › Supplementary Information Figure S1. BUSCO.jpg]
